# Supplementary material for: OTUD6A promotes prostate tumorigenesis via deubiquitinating Brg1 and AR
Source: Commun Biol. 2022 Mar 1;5:182. doi: 10.1038/s42003-022-03133-1 (PMC8888634; doi:10.1038/s42003-022-03133-1)

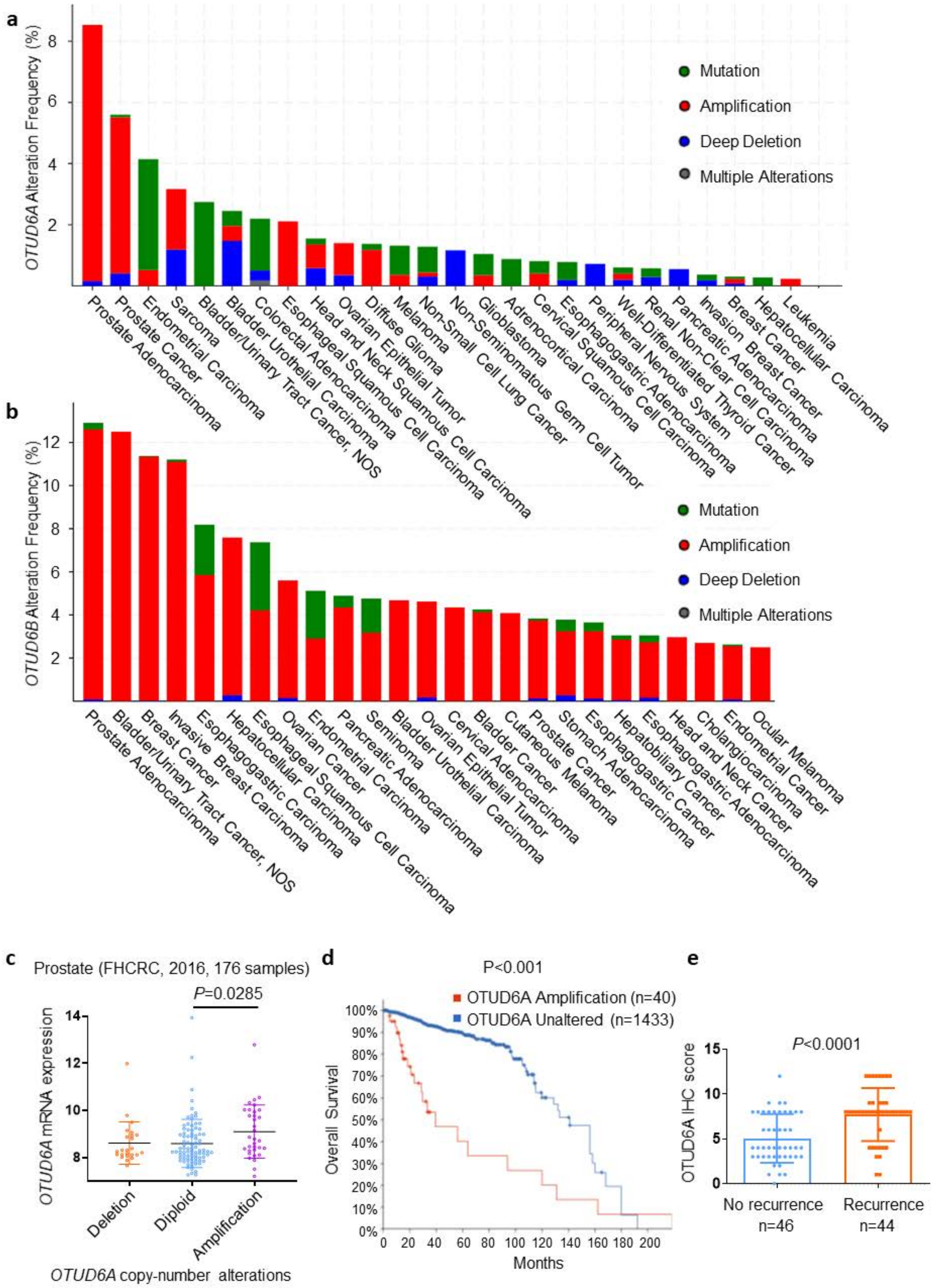

Supplementary Figure 1: OTUD6A is amplified in PCa

a, b The alteration frequency (including mutation, amplification, deep deletion and multiple alterations) of OTUD6A (a) and OTUD6B (b) in different cancer patient samples were analyzed by integrating the online datasets (http://www.cbioportal.org/).

c The correlation of *OTUD6A* DNA copy number alteration with *OTUD6A* mRNA expression levels in PCa patient samples in the FHCRC (2016, 176 samples) dataset (http://www.cbioportal.org/). **d** Kaplan-Meier plot of overall survival based on the OTUD6A amplification or unaltered in PCa patient samples by analyzing the integrated online datasets (http://www.cbioportal.org/). **e** The correlation of OTUD6A expression with biochemical recurrence in PCa patients from the Shanghai Changhai Hospital cohort (n=90).

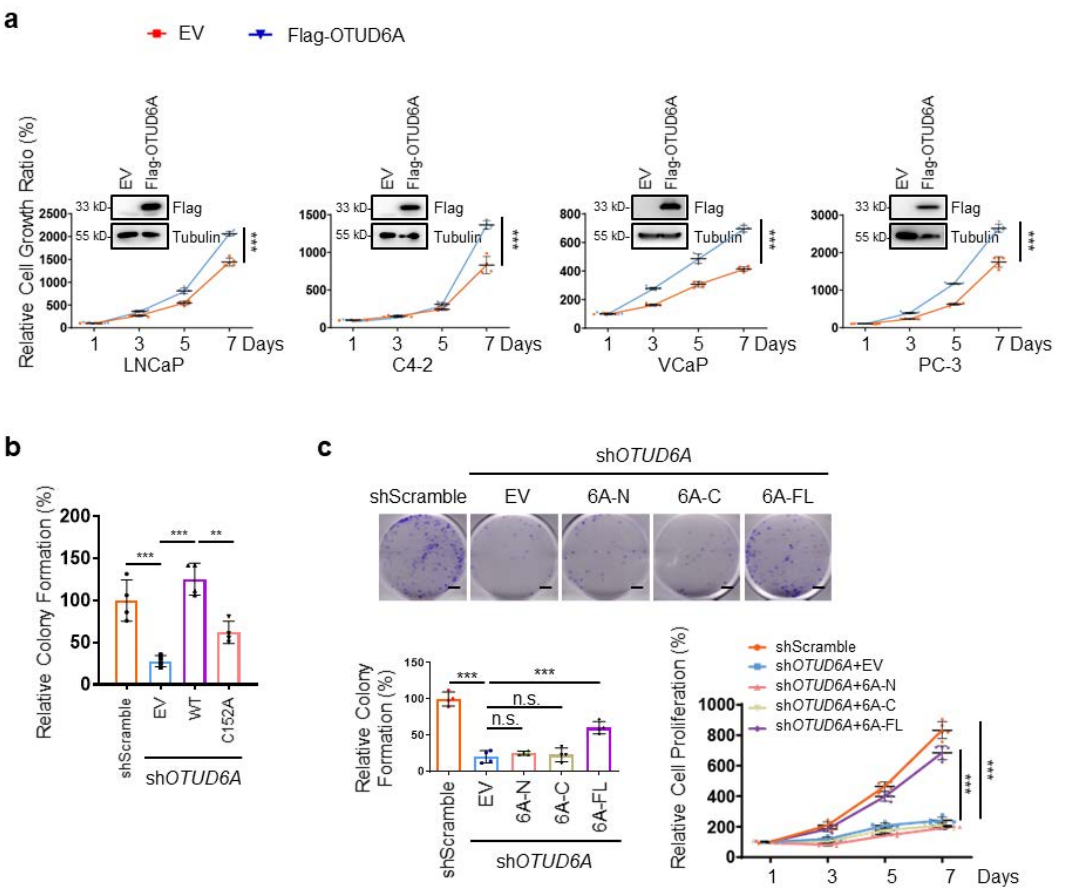

Supplementary Figure 2: OTUD6A promotes PCa proliferation

a LNCaP, C4-2, VCaP and PC-3 cells were transfected with empty vector (EV) or Flag-OTUD6A plasmids and cell growth ratio were examined by SRB assays. Data were shown as mean ± SD of three independent experiments. ***P<0.001. Immunoblotting (IB) analysis of the whole cell lysates (WCLs) were shown on the top of curves. **b** The statistic results related to Fig. 2d. Data were shown as mean ± SD of three independent experiments. **0.001<P<0.01, ***P<0.001, n.s.: non-significant. **c** Representative images of the colony formation assay of C4-2-shScramble or C4-2-sh*OTUD6A* cells transfected with the indicated plasmids. Scale bar: 5 mm. The statistic results were shown as mean ± SD of three independent experiments. And the corresponding cell growth ratio were examined by SRB assays (right panel). Data were shown as mean ±

SD of three independent experiments. ***P<0.001. 6A-N, N-terminus of OTUD6A; 6A-C, C-terminus of OTUD6A; 6A-FL, full length of OTUD6A.

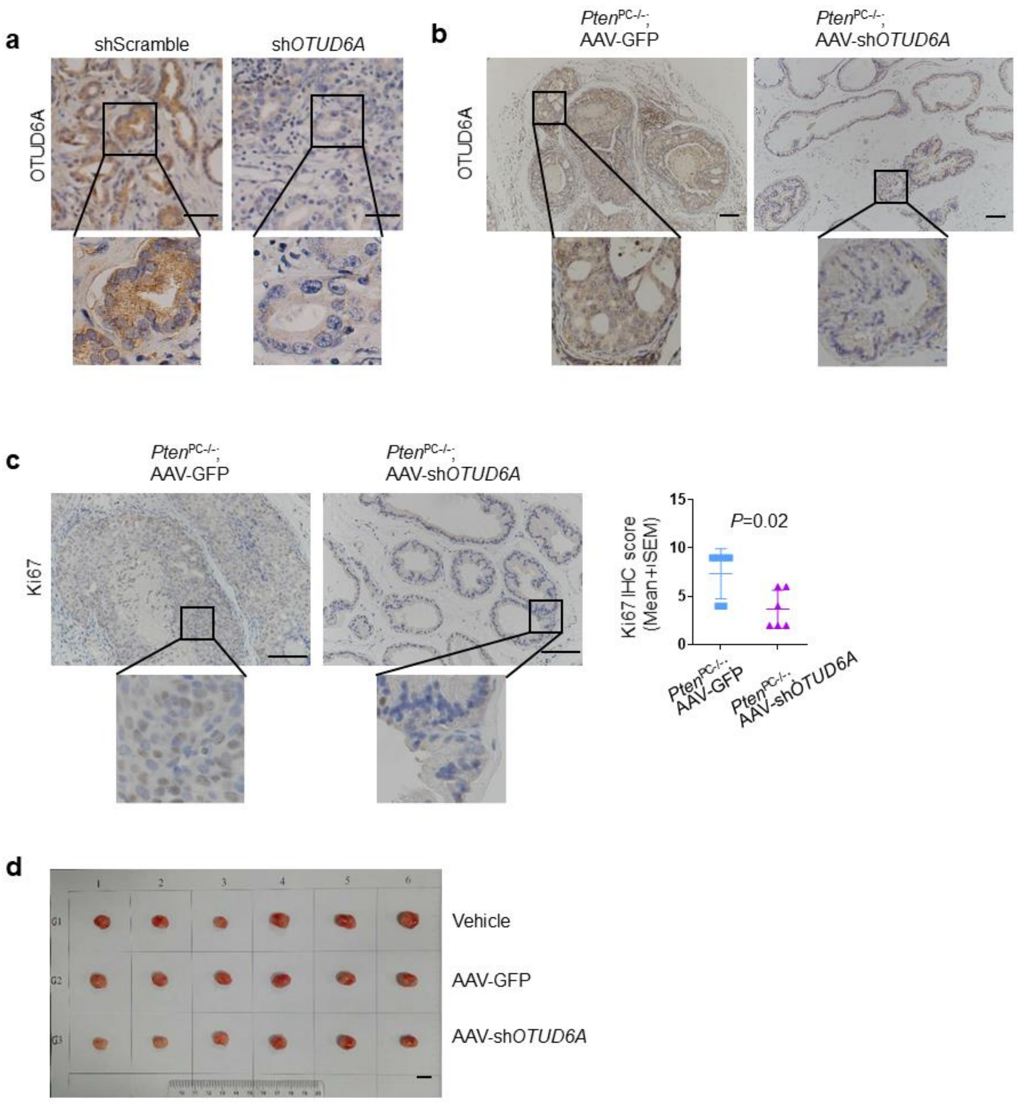

Supplementary Figure 3: OTUD6A oligonucleotides markedly suppress prostate tumorigenesis derived from Pten$^{PC-/-}$ mice and patient-derived xenograft (PDX) models

a Representative images of OTUD6A IHC staining of C4-2-derived xenografts. Scale bars: 50 μm. **b** OTUD6A-stained sections of representative VP tissues, scale bars: 100 μm. **c** Representative images and scores of Ki67 IHC staining in AAV-GFP and AAV-sh*OTUD6A* treatment groups. Scale bar: 100 μm. Data were shown as mean ± SD. Statistical significance was determined by two-tailed unpaired Student's t-test. **d** Image of the collected tumors related to Fig. 3e-g. Scale bar: 1 cm.

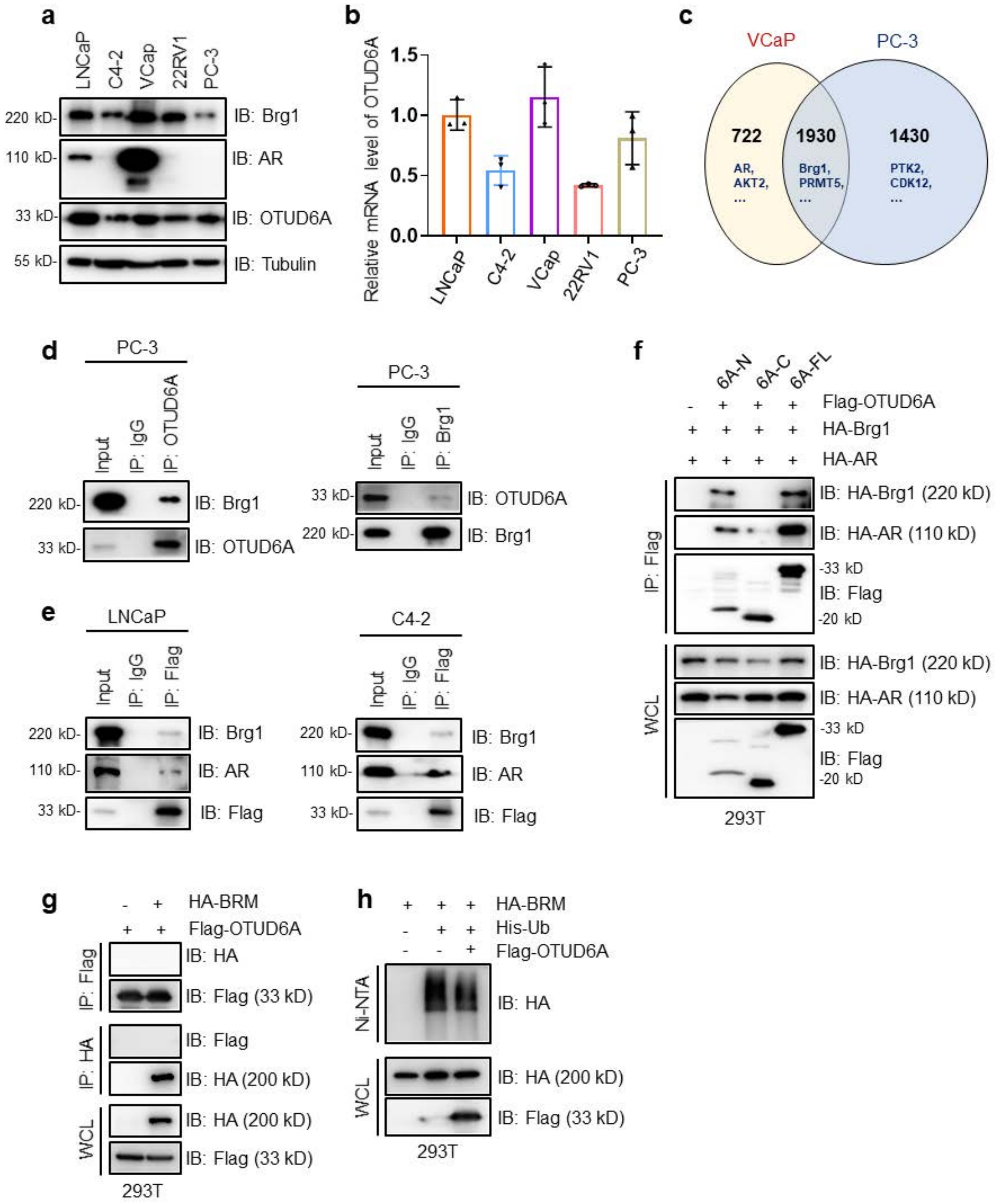

Supplementary Figure 4: OTUD6A binds with Brg1 and AR

a The protein expression of OTUD6A, Brg1 and AR in different PCa cell lines were examined by IB. **b** The mRNA expression of OTUD6A in different PCa cell lines were examined by RT-PCR. **c** Venn diagram showed the number of identified OTUD6A interaction proteins in VCaP and PC-3 cells by IP-MS. **d** Co-IP experiments in PC-3 cells were performed using anti-OTUD6A (left) or anti-Brg1 (right) antibodies. IgG was used as a negative control. **e** Co-IP experiments in LNCaP and C4-2 cells

transfected with Flag-OTUD6A were performed using anti-Flag antibody or anti-mouse IgG. **f** IB analysis of WCLs and IPs derived from 293T cells transfected with Flag-OTUD6A fragments together with the indicated HA-Brg1 and HA-AR plasmids. 6A-N, N-terminus of OTUD6A; 6A-C, C-terminus of OTUD6A; 6A-FL, full length of OTUD6A. **g** IB analysis of WCLs and IPs derived from 293T cells transfected with Flag-OTUD6A together with or without HA-BRM. **h** IB analysis of WCLs and IPs derived from 293T cells transfected with the indicated HA-BRM, Flag-OTUD6A and His-Ub plasmids.

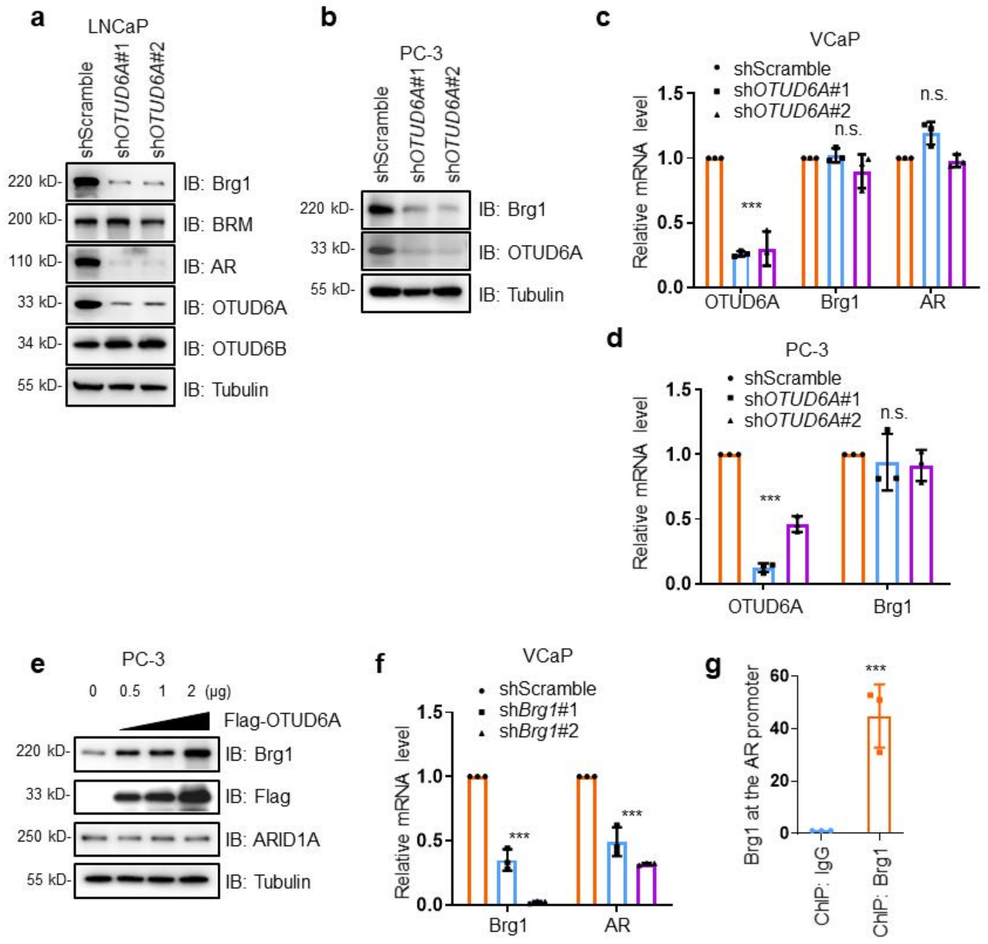

Supplementary Figure 5: OTUD6A stabilizes Brg1 and AR

a IB analysis in LNCaP cells infected with lentivirus expressing indicated shRNAs. **b** IB analysis in PC-3 cells infected with lentivirus expressing indicated shRNAs. **c, d** VCaP and PC-3 cells were infected with lentivirus expressing indicated shRNAs and then for RT-PCR analysis. Data were shown as mean ± SD (n = 3), ***P<0.001, n.s.: non-significant. **e** IB analysis in PC-3 cells transfected with the indicated plasmids for 48 hr. **f** VCaP cells were infected with lentivirus expressing indicated shRNAs and then for RT-PCR analysis. Data were shown as mean ± SD (n = 3), ***P<0.001. **g** The ChIP assay was carried out using antibodies directed against IgG or Brg1 in VCaP cells, and then the bound and input DNAs were analyzed using primers of *AR* promoter region by

RT-PCR. IgG was used as a negative control. Data were shown as mean ± SD (n = 3), $^{***}P<0.001$.

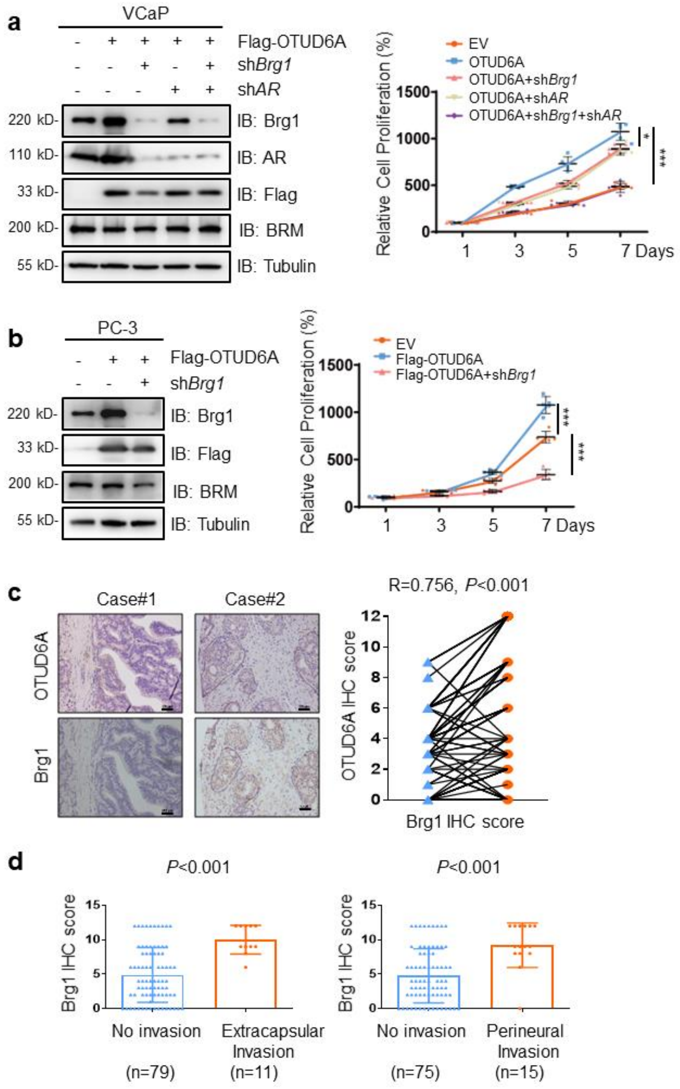

Supplementary Figure 6: Brg1 expression is highly correlated with OTUD6A expression and tumor progress in PCa

a, b VCaP and PC-3 cells were transfected with indicated plasmids and cell growth ratio were examined by SRB assays. Data were shown as mean ± SD of three independent experiments. *P<0.05, ***P<0.001. The left panels showed the corresponding IB results. **c** Representative images of OTUD6A and Brg1 IHC staining in prostate cancer tissues. And the right panel showed the correlation of OTUD6A and Brg1. n=90; Scale bar, 100 μm. **d** The correlation of Brg1 with extracapsular invasion and perineural invasion in PCa patient samples (n=90).

Supplementary Figure 7

Uncropped blots related to Figure 2a

OTUD6A (C4-2) (LNCaP) (VCaP) (PC-3)

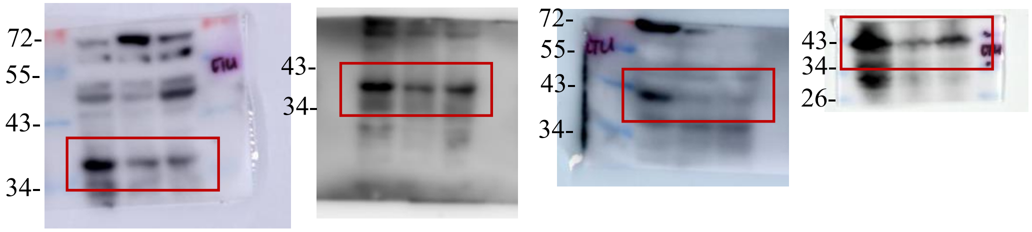

OTUD6B (LNCaP) (C4-2) (VCaP) (PC-3)

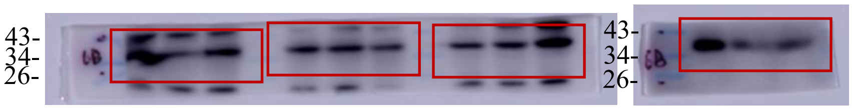

Tubulin (C4-2) (LNCaP) (VCaP) (PC-3)

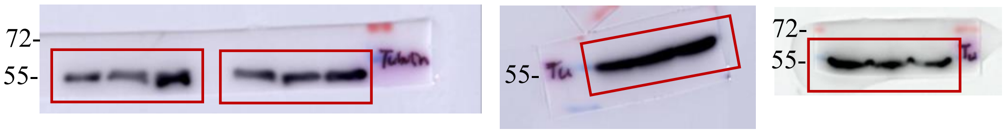

Uncropped blots related to Figure 2c

OTUD6A

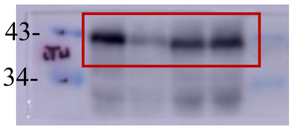

Tubulin

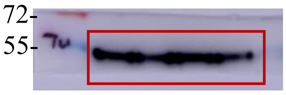

Uncropped blots related to Figure 3d, 3h, 4b

Fig. 3d-PTEN

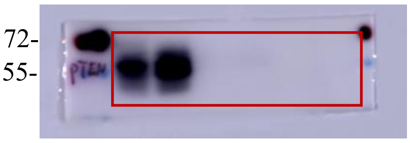

Fig. 3d-OTUD6A

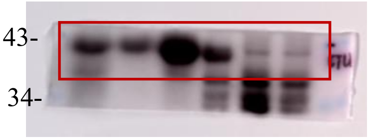

Fig. 3d-Tubulin

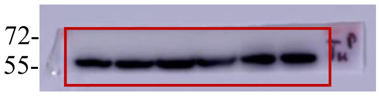

Fig. 3h-OTUD6A

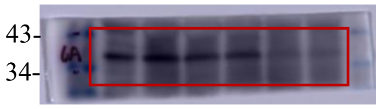

Fig. 3h-Tubulin

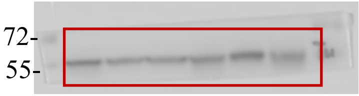

Fig. 4b-Brg1 (upper)

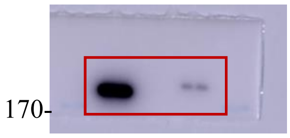

Fig. 4b-AR (upper)

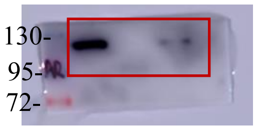

Fig. 4b-OTUD6A (upper)

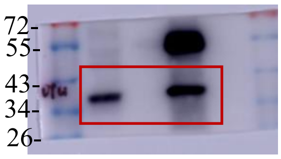

Fig. 4b-OTUD6A (lower)

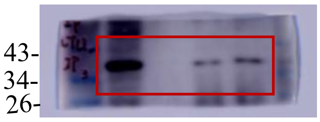

Fig. 4b-AR (lower)

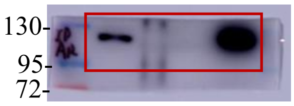

Fig. 4b-Brg1 (lower)

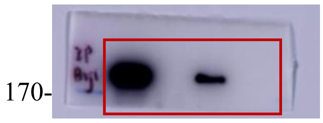

Uncropped blots related to Figure 4c, 4d

Fig. 4c-HA-Brg1 (IP)

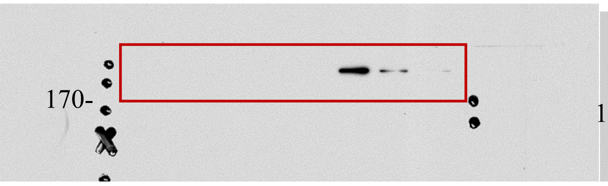

Fig. 4c-HA-Brg1 (WCL)

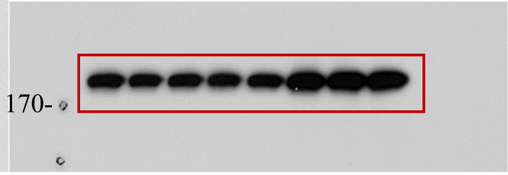

Fig. 4c-Flag-DUBs (IP)

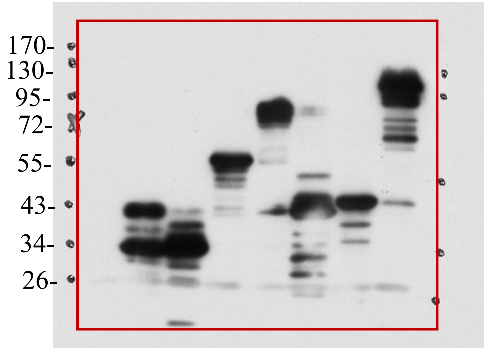

Fig. 4c-Flag-DUBs (WCL)

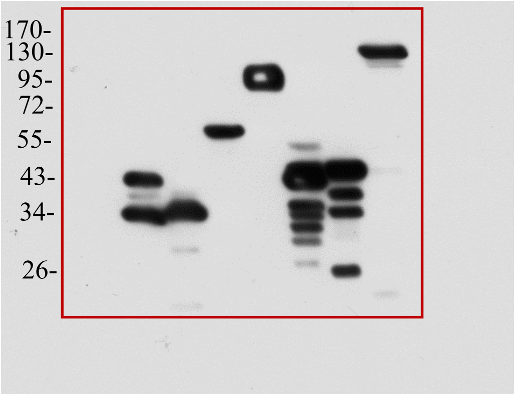

Fig. 4d-HA-AR (IP)

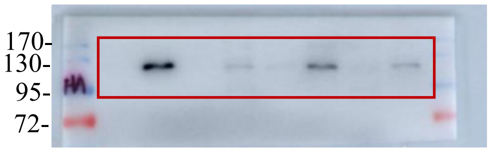

Fig. 4d-HA-AR (WCL)

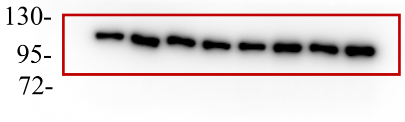

Fig. 4d-Flag-DUBs (IP)

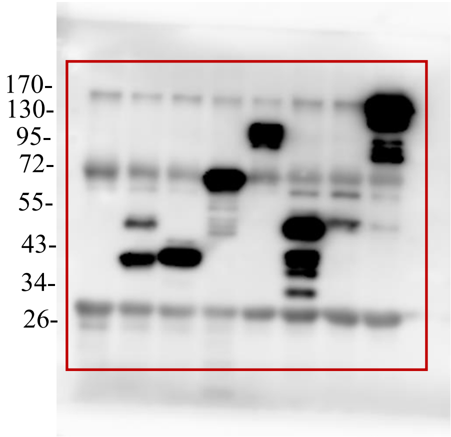

Fig. 4d-Flag-DUBs (WCL)

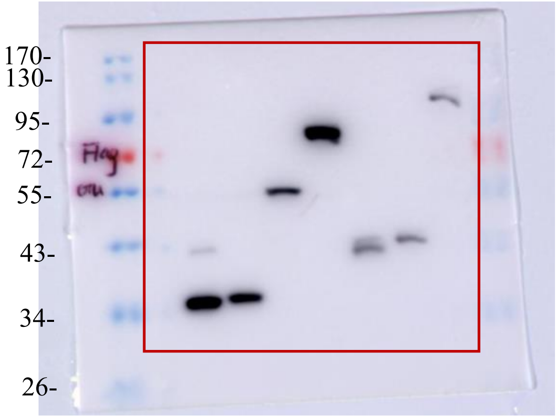

Uncropped blots related to Figure 4e

Brg1

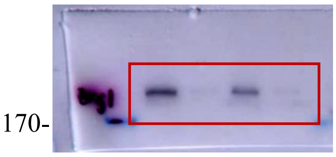

AR

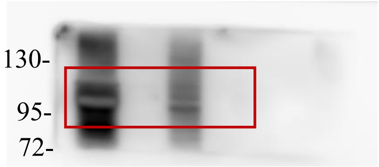

Input

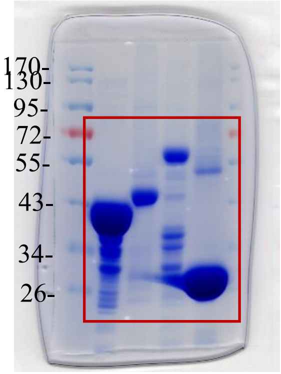

Uncropped blots related to Figure 4f, 4g

Fig. 4f-HA-Brg1 (IP)

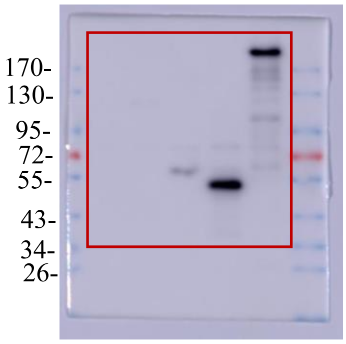

Fig. 4f-HA-Brg1 (Input)

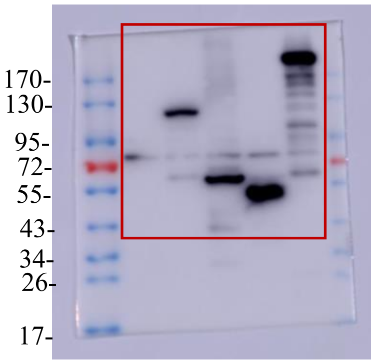

Fig. 4f-Flag-OTUD6A (IP)

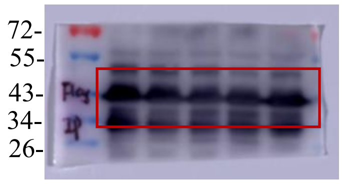

Fig. 4f-Flag-OTUD6A (Input)

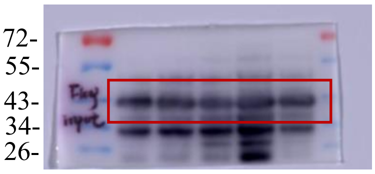

Fig. 4g-HA-AR (IP)

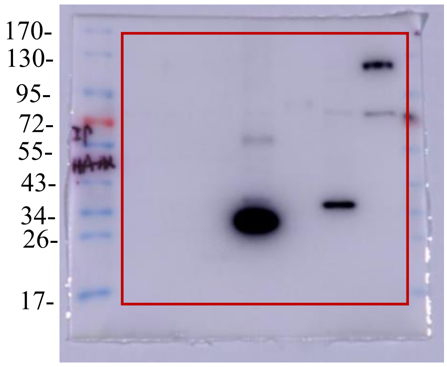

Fig. 4g-HA-AR (Input)

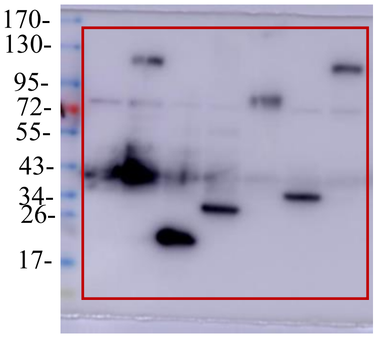

Fig. 4g-Flag-OTUD6A (IP)

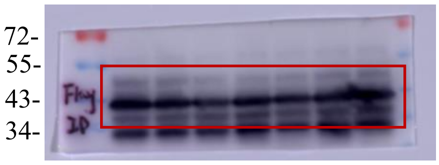

Fig. 4g-Flag-OTUD6A (Input)

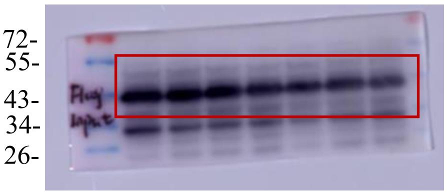

Uncropped blots related to Figure 5a

Brg1

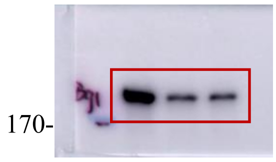

BRM

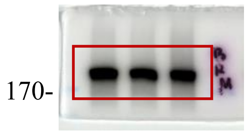

AR

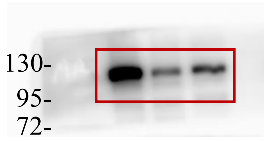

OTUD6A

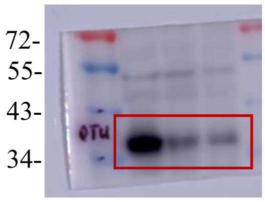

OTUD6B

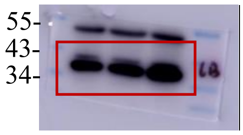

Tubulin

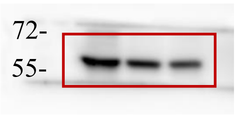

Uncropped blots related to Figure 5b

Brg1 (VCaP)

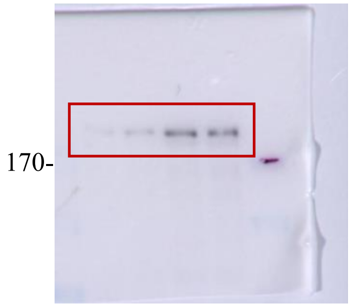

HA-Brg1 (293T)

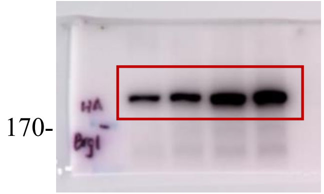

BRM (VCaP)

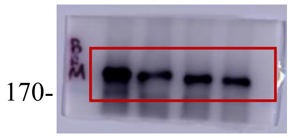

AR (VCaP)

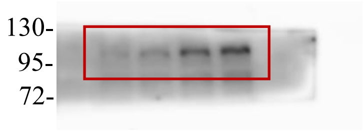

HA-AR (293T)

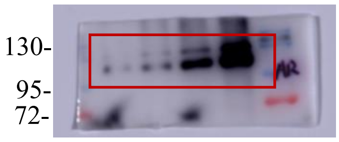

Flag-OTUD6A (VCaP)

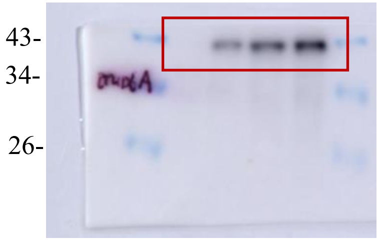

Flag-OTUD6A (293T)

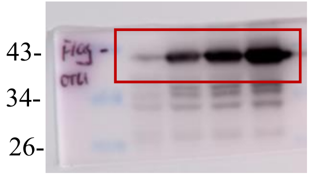

Tubulin (VCaP)

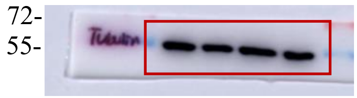

Tubulin (293T)

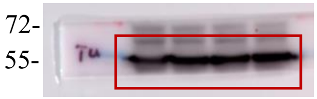

Uncropped blots related to Figure 5c, 5d

Fig. 5c-AR (shOTUD6A)

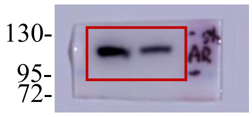

Fig. 5c-AR (Flag-OTUD6A)

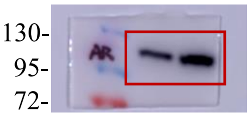

Fig. 5c-OTUD6A (shOTUD6A)

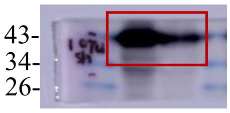

Fig. 5c-OTUD6A (Flag-OTUD6A)

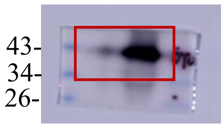

Fig. 5c-Tubulin (shOTUD6A)

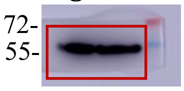

Fig. 5c- Tubulin (Flag-OTUD6A)

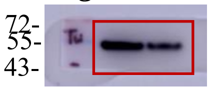

Fig. 5d-Brg1

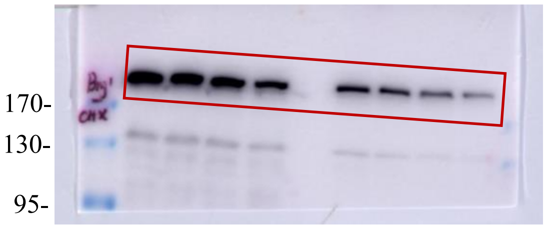

Fig. 5d-AR

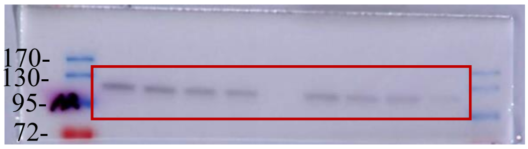

Fig. 5d-OTUD6A

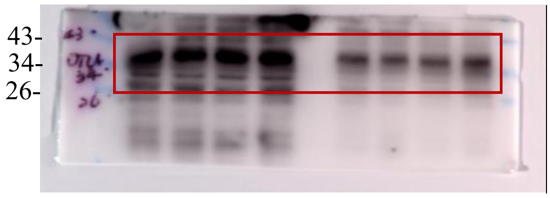

Fig. 5d-Tubulin

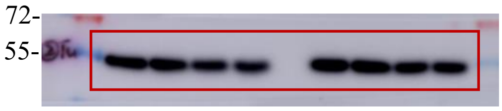

Uncropped blots related to Figure 5e, 5f

Fig.5e-Brg1 (IP: HA)

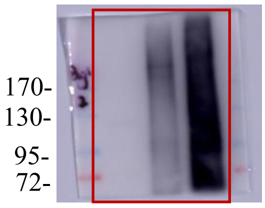

Fig.5e-AR (IP: HA)

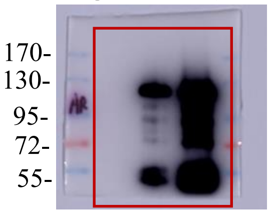

Fig.5e-Brg1 (IP: WCL)

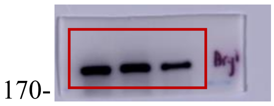

Fig.5e-AR (WCL)

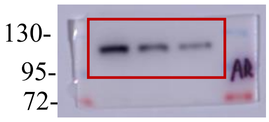

Fig.5e-OTUD6A (WCL)

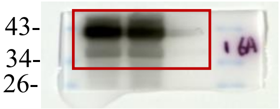

Fig.5e-Tubulin (WCL)

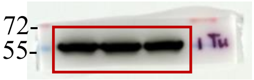

Fig.5f-AR (IP: HA)

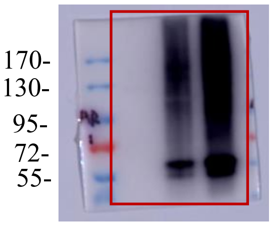

Fig.5f-OTUD6A (WCL)

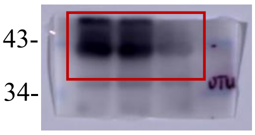

Fig.5f-AR (WCL)

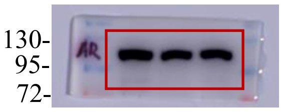

Fig.5f-Tubulin (WCL)

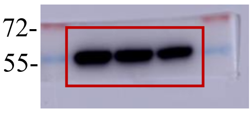

Uncropped blots related to Figure 5g

HA-Brg1 (Ni-NTA)

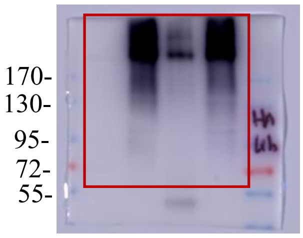

HA-AR (Ni-NTA)

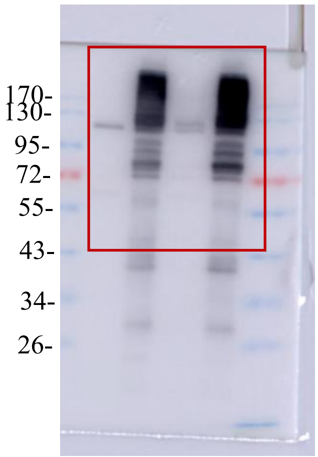

HA-Brg1 (WCL)

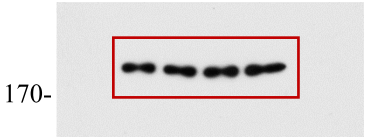

HA-AR (WCL)

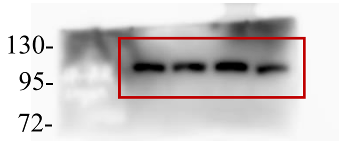

Flag-OTUD6A/OTUD6B (WCL)

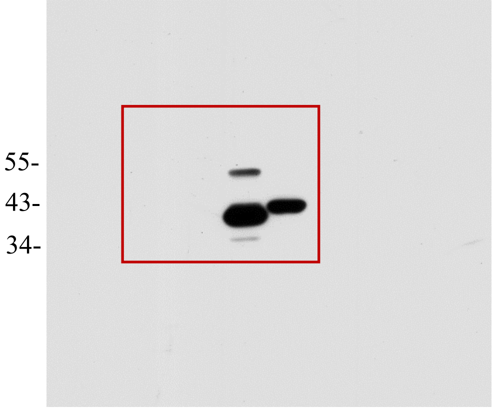

Flag-OTUD6A/OTUD6B (WCL)

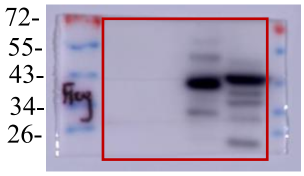

Uncropped blots related to Figure 5h

HA-Brg1 (Ni-NTA)

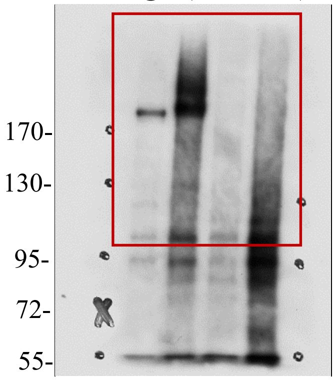

HA-AR (Ni-NTA)

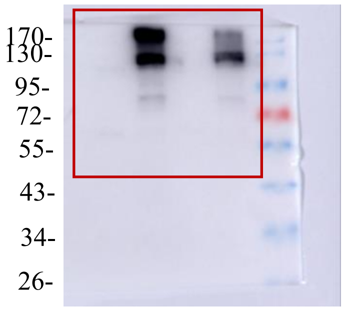

HA-Brg1 (WCL)

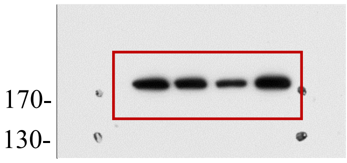

HA-AR (WCL)

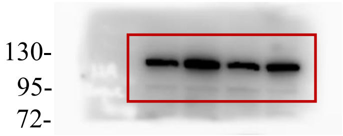

Flag-OTUD6A (WCL)

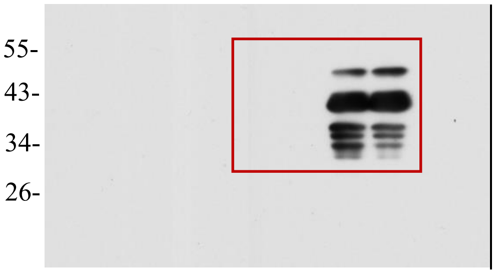

Flag-OTUD6A (WCL)

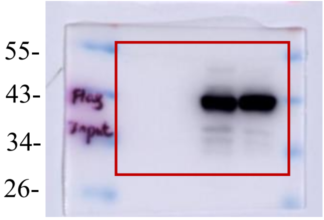

Uncropped blots related to Figure 5i

Brg1

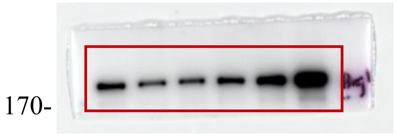

AR

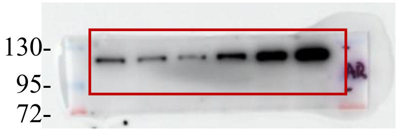

Flag-OTUD6A

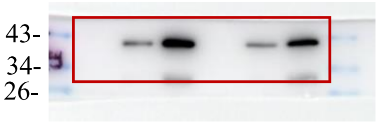

Tubulin

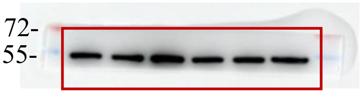

Uncropped blots related to Figure 6a, 6b

Fig. 6a-HA (Ub)n-Brg1 (IP: Flag)

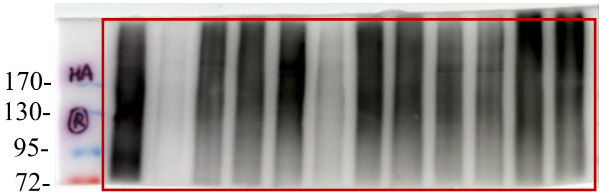

Fig. 6a-Myc-OTUD6A (Input)

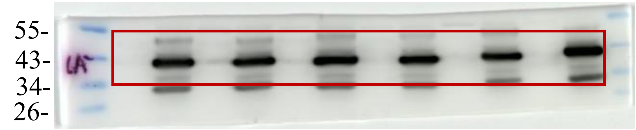

Fig. 6a-Flag-Brg1 (Input)

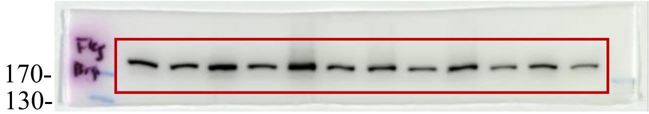

Fig. 6b-HA (Ub)n-Brg1 (IP: Flag)

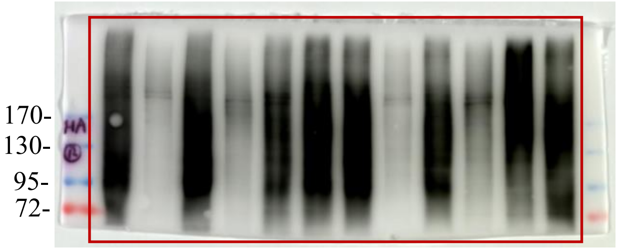

Fig. 6b-Myc-OTUD6A (Input)

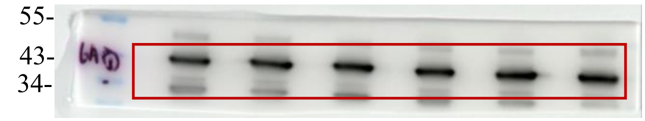

Fig. 6b-Flag-Brg1 (Input)

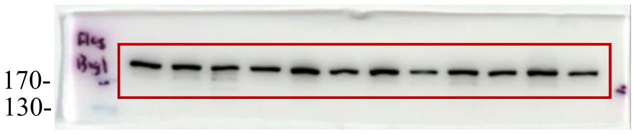

Uncropped blots related to Figure 6c, 6d

Fig. 6c-HA (Ub)n-AR (IP: Flag)

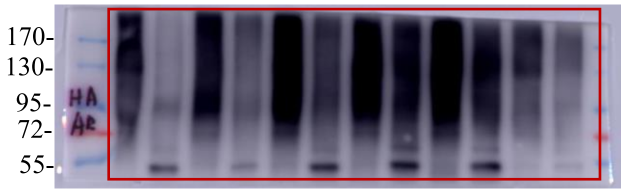

Fig. 6c-Myc-OTUD6A (Input)

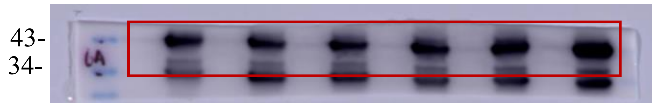

Fig. 6c-Flag-AR (Input)

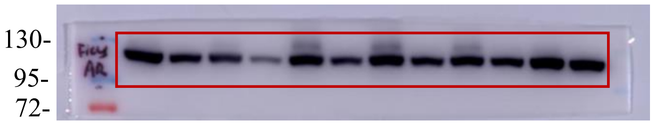

Fig. 6d-HA (Ub)n-AR (IP: Flag)

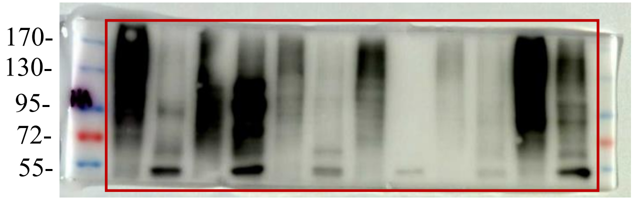

Fig. 6d-Myc-OTUD6A (Input)

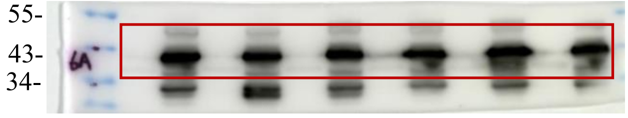

Fig. 6d-Flag-AR (Input)

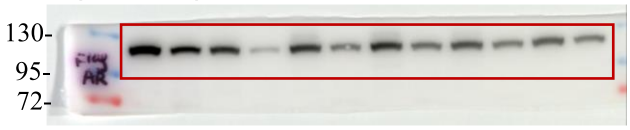

Uncropped blots related to Figure 6e, 6f, 6g

Fig. 6e-HA (Ni-NTA)

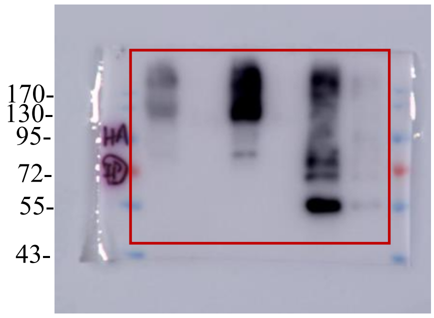

Fig. 6e-Flag-OTUD6A (WCL)

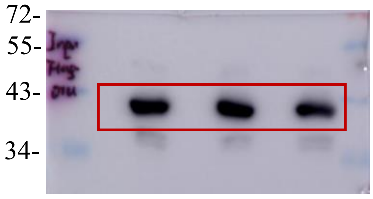

Fig. 6e-GFP-FBW7 (WCL)

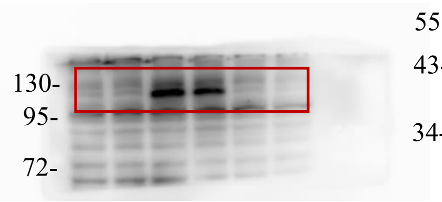

Fig. 6e-Myc-SPOP (WCL)

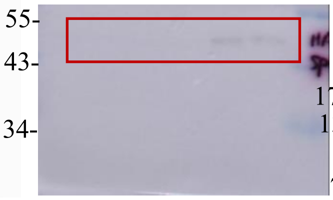

Fig. 6e-HA-Brg1/AR (WCL)

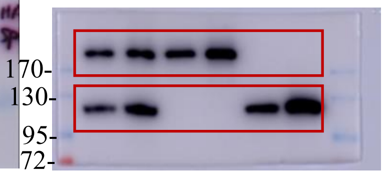

Fig. 6f-HA (Ub)n-Brg1 (IP)

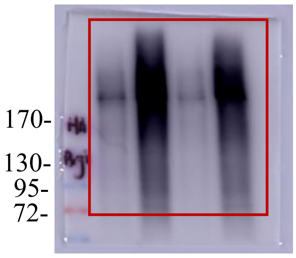

Fig. 6g-HA (Ub)n-AR (IP)

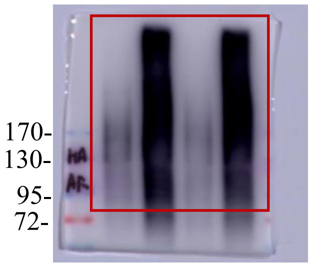

Fig. 6f-GFP-FBW7 (Input)

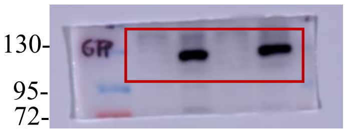

Fig. 6g-Myc-SPOP (Input)

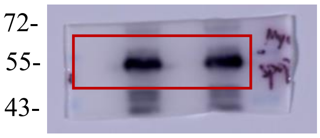

Fig. 6f-Flag-Brg1 (Input)

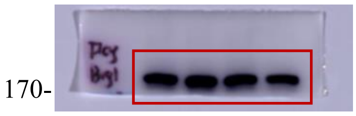

Fig. 6g-Flag-AR (Input)

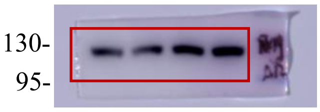

Uncropped blots related to Supplementary Figure 2a, 4a

Fig. S2a-Flag-OTUD6A (LNCaP) (C4-2) (VCaP) (PC-3)

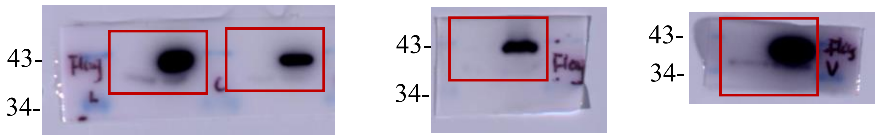

Fig. S2a-Tubulin (LNCaP) (C4-2) (VCaP) (PC-3)

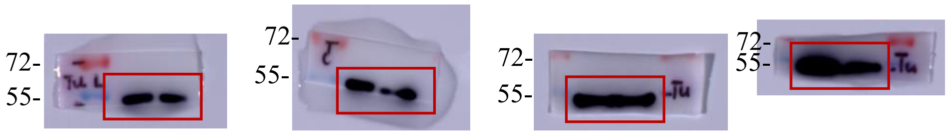

Fig. S4a-Brg1

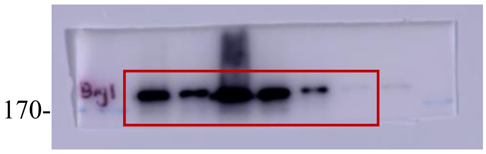

Fig. S4a-AR

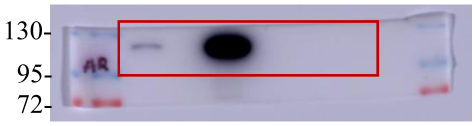

Fig. S4a-OTUD6A

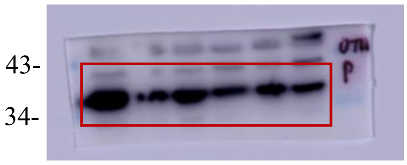

Fig. S4a-Tubulin

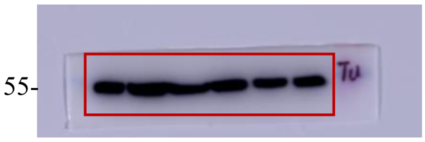

Uncropped blots related to Supplementary Figure 4d, 4e

Fig. S4d-Brg1 (IP: OTUD6A) (PC-3)

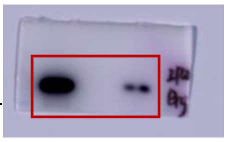

170-

Fig. S4d-OTUD6A (IP: OTUD6A) (PC-3)

72-
55-
43-
34-

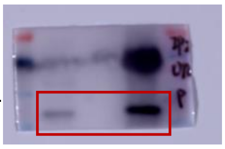

Fig. S4d-OTUD6A (IP: Brg1) (PC-3)

72-
55-
43-
34-

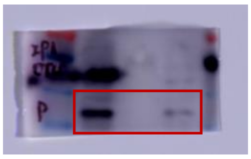

Fig. S4d-Brg1 (IP: Brg1) (PC-3)

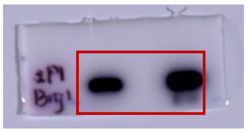

170-

Fig. S4e-Brg1 (LNCaP)

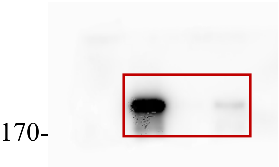

170-

Fig. S4e-Brg1 (C4-2)

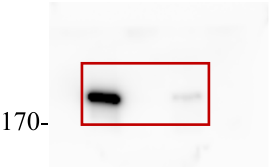

170-

Fig. S4e-AR (LNCaP)

130-
95-
72-

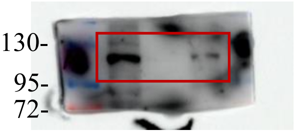

Fig. S4e-AR (C4-2)

130-
95-
72-

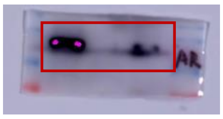

Fig. S4e-Flag-OTUD6A (LNCaP)

72-
55-
43-
34-

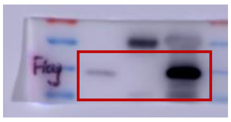

Fig. S4e-Flag-OTUD6A (C4-2)

72-
55-
43-
34-

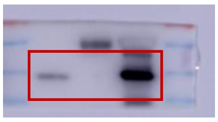

Uncropped blots related to Supplementary Figure 4f, 4g, 4h

Fig. S4f-HA-Brg1 (IP)

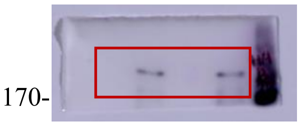

Fig. S4f-HA-Brg1 (Input)

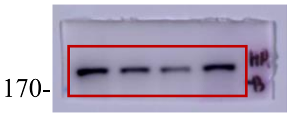

Fig. S4f-HA-AR (IP)

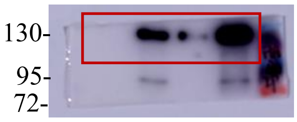

Fig. S4f-HA-AR (Input)

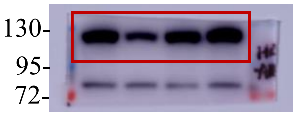

Fig. S4f-Flag-OTUD6A (IP)

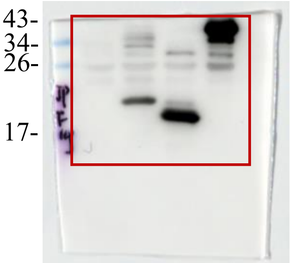

Fig. S4f-Flag-OTUD6A (Input)

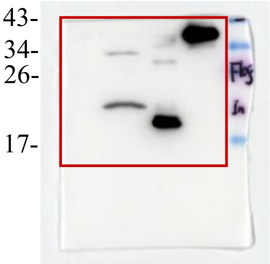

Fig. S4g-HA-BRM (IP: WCL, IP: Flag, IP: HA)

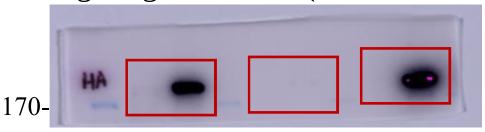

Fig. S4g-Flag-OTUD6A (WCL, IP: Flag, IP: HA)

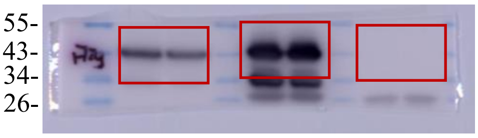

Fig. S4h-HA-BRM (Ni-NTA)

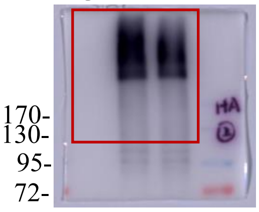

Fig. S4h-HA-BRM (WCL)

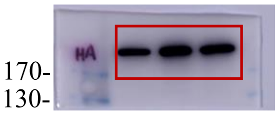

Fig. S4h-Flag-OTUD6A (WCL)

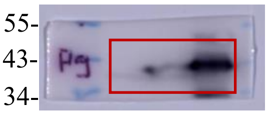

Uncropped blots related to Supplementary Figure 5a, 5b, 5e

Fig. S5a-Brg1

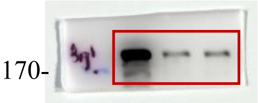

Fig. S5a-BRM

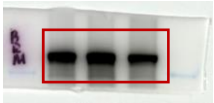

Fig. S5a-AR

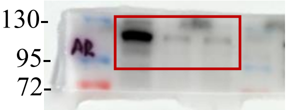

Fig. S5a-OTUD6A

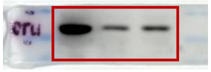

Fig. S5a-OTUD6B

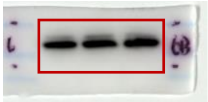

Fig. S5a-Tubulin

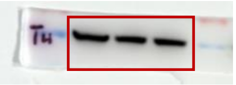

Fig. S5b-Brg1

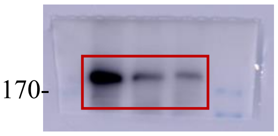

Fig. S5b-OTUD6A

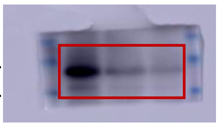

Fig. S5b-Tubulin

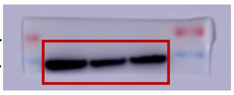

Fig. S5e-Brg1

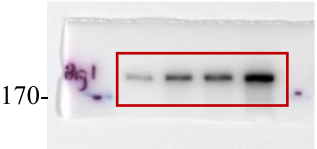

Fig. S5e-Flag-OTUD6A

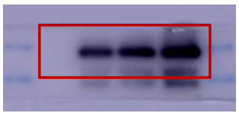

Fig. S5e-ARID1A

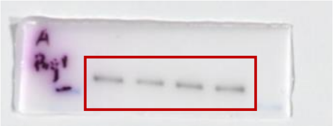

Fig. S5e-Tubulin

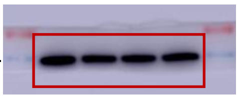

Uncropped blots related to Supplementary Figure 6a, 6b

Fig. S6a-Brg1

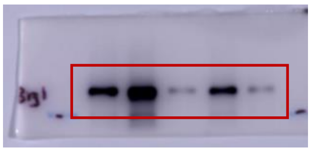

Fig. S6a-AR

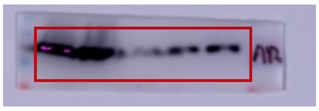

Fig. S6a-Flag-OTUD6A

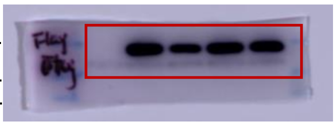

Fig. S6a-BRM

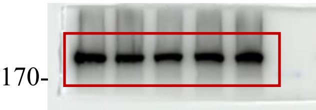

Fig. S6a-Tubulin

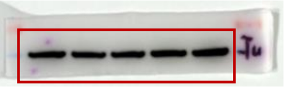

Fig. S6b-Brg1

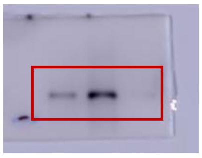

Fig. S6b-Flag-OTUD6A

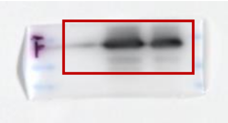

Fig. S6b-BRM

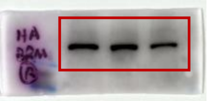

Fig. S6b-Tubulin

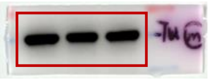

Supplement: Supplementary file 2 — Supplementary Information [file 42003_2022_3133_MOESM2_ESM.pdf]
