# Supplementary material for: OTUD6A promotes prostate tumorigenesis via deubiquitinating Brg1 and AR
Source: Commun Biol. 2022 Mar 1;5:182. doi: 10.1038/s42003-022-03133-1 (PMC8888634; doi:10.1038/s42003-022-03133-1)
Supplement: Supplementary file 3 — Description of Additional Supplementary Files [file 42003_2022_3133_MOESM3_ESM.pdf]

Description of Additional Supplementary Files

File name: Supplementary Data 1
Description: Patients for IHC, Related to Figure 1.

File name: Supplementary Data 2
Description: MS, Related to Figure 4.

File name: Supplementary Data 3
Description: RNA-seq, Related to Figure 6.

File name: Supplementary Data 4
Description: Source data.
