# Supplementary material for: OTUD6A promotes prostate tumorigenesis via deubiquitinating Brg1 and AR
Source: Commun Biol. 2022 Mar 1;5:182. doi: 10.1038/s42003-022-03133-1 (PMC8888634; doi:10.1038/s42003-022-03133-1)
Supplement: Supplementary file 8 — Reporting Summary [file 42003_2022_3133_MOESM8_ESM.pdf]

nature portfolio

Corresponding author(s): Liyu Huang

Last updated by author(s): Dec 15, 2021

Reporting Summary

Nature Portfolio wishes to improve the reproducibility of the work that we publish. This form provides structure for consistency and transparency in reporting. For further information on Nature Portfolio policies, see our Editorial Policies and the Editorial Policy Checklist.

Statistics

For all statistical analyses, confirm that the following items are present in the figure legend, table legend, main text, or Methods section.

n/a	Confirmed	
☐	☒	The exact sample size (*n*) for each experimental group/condition, given as a discrete number and unit of measurement
☐	☒	A statement on whether measurements were taken from distinct samples or whether the same sample was measured repeatedly
☐	☒	The statistical test(s) used AND whether they are one- or two-sided<br>*Only common tests should be described solely by name; describe more complex techniques in the Methods section.*
☐	☒	A description of all covariates tested
☒	☐	A description of any assumptions or corrections, such as tests of normality and adjustment for multiple comparisons
☐	☒	A full description of the statistical parameters including central tendency (e.g. means) or other basic estimates (e.g. regression coefficient) AND variation (e.g. standard deviation) or associated estimates of uncertainty (e.g. confidence intervals)
☐	☒	For null hypothesis testing, the test statistic (e.g. *F*, *t*, *r*) with confidence intervals, effect sizes, degrees of freedom and *P* value noted<br>*Give P values as exact values whenever suitable.*
☒	☐	For Bayesian analysis, information on the choice of priors and Markov chain Monte Carlo settings
☒	☐	For hierarchical and complex designs, identification of the appropriate level for tests and full reporting of outcomes
☒	☐	Estimates of effect sizes (e.g. Cohen's *d*, Pearson's *r*), indicating how they were calculated

Our web collection on statistics for biologists contains articles on many of the points above.

Software and code

Policy information about availability of computer code

Data collection	http://www.cbioportal.org/<br>http://david.abcc.ncifcrf.gov
Data analysis	We used the ImageJ software to quantify the protein bands intensity and used the GraphPad Prism 8 and Excel to do the graph figures and statistics.

For manuscripts utilizing custom algorithms or software that are central to the research but not yet described in published literature, software must be made available to editors and reviewers. We strongly encourage code deposition in a community repository (e.g. GitHub). See the Nature Portfolio guidelines for submitting code & software for further information.

Data

Policy information about availability of data

All manuscripts must include a data availability statement. This statement should provide the following information, where applicable:

- Accession codes, unique identifiers, or web links for publicly available datasets
- A description of any restrictions on data availability
- For clinical datasets or third party data, please ensure that the statement adheres to our policy

The RNA-Seq data has been deposited to the National Genomics Data Center (NGDC) (BioProject: PRJCA007053). Data pertaining to TCGA prostate cancer samples were obtained from the cBioPortal. Additional information is avalible from the authors upon reasonable request.

Field-specific reporting

Please select the one below that is the best fit for your research. If you are not sure, read the appropriate sections before making your selection.

☒ Life sciences ☐ Behavioural & social sciences ☐ Ecological, evolutionary & environmental sciences

For a reference copy of the document with all sections, see nature.com/documents/nr-reporting-summary-flat.pdf

Life sciences study design

All studies must disclose on these points even when the disclosure is negative.

Sample size	We determined sample size based on the sufficient statistics. For IHC experiments, 90 prostate cancer patient samples from Shanghai Changhai Hospital were used. For mice in vivo assays, the numbers of mice (n=6) were included in the figure legends.
Data exclusions	No data was excluded from the experiments.
Replication	Yes, the experimental findings were reliably reproduced.
Randomization	Samples were allocated into each group randomly in this study.
Blinding	The investigators were blinded to group allocation during data acquisition and analysis.

Reporting for specific materials, systems and methods

We require information from authors about some types of materials, experimental systems and methods used in many studies. Here, indicate whether each material, system or method listed is relevant to your study. If you are not sure if a list item applies to your research, read the appropriate section before selecting a response.

Materials & experimental systems

n/a	Involved in the study	
☐	☒	Antibodies
☐	☒	Eukaryotic cell lines
☒	☐	Palaeontology and archaeology
☐	☒	Animals and other organisms
☒	☐	Human research participants
☒	☐	Clinical data
☒	☐	Dual use research of concern

Methods

n/a	Involved in the study	
☒	☐	ChIP-seq
☒	☐	Flow cytometry
☒	☐	MRI-based neuroimaging

Antibodies

Antibodies used	OTUD6A (Invitrogen, PA5-62772; Proteintech, 24486-1-AP), Brg1 (CST, #72182), AR (Santa Cruz, sc-7305), PTEN (CST, #9556), HA (Proteintech, 51064-2AP), Flag (Sigma, F3165), GFP (Proteintech, 66002-1-Ig), Ub (Santa Cruz, sc-8017), β-Tubulin (BPI, AbM59005-37B-PU)
Validation	All primary antibodies used in this study are commercially available and have been validated by the manufactures.

Eukaryotic cell lines

Policy information about cell lines

Cell line source(s)	Human prostate cancer cell lines PC-3 and C4-2 were obtained from the Institute of Biochemistry and Cell Biology (Chinese Academy of Sciences); VCaP and LNCaP cells were kindly provided by Cell Bank, Chinese Academy of Sciences. Mouse prostate cancer cell line Myc-CaP was obtained from Dr. Xuanming Yang (School of Life Sciences and Biotechnology, Shanghai Jiao Tong University); Human embryonic kidney 293 (HEK293T) cells were obtained from Dr. Wenyi Wei (Harvard Medical School, MA).
Authentication	All cells were authenticated by STR (Short Tandem Repeat).
Mycoplasma contamination	All cell lines tested negative for mycoplasma contamination.
Commonly misidentified lines (See ICLAC register)	Not applicable.

Animals and other organisms

Policy information about studies involving animals; ARRIVE guidelines recommended for reporting animal research

Laboratory animals	NOD/SCID male mice (6 weeks old)<br>FVB male mice (6 weeks old)<br>NOG/SCID male mice (6 weeks old)<br>C57BL/6 female and male mice (all ages)
Wild animals	Not applicable.
Field-collected samples	The study did not involve samples collected from the field.
Ethics oversight	Study protocols involving mice were approved by the Institutional Animal Care and Use Committees (IACUC) of Shanghai JiaoTong University.

Note that full information on the approval of the study protocol must also be provided in the manuscript.
